# A chimeric haemagglutinin-based influenza split virion vaccine adjuvanted with AS03 induces protective stalk-reactive antibodies in mice

**DOI:** 10.1038/npjvaccines.2016.15

**Published:** 2016-09-22

**Authors:** Raffael Nachbagauer, David Kinzler, Angela Choi, Ariana Hirsh, Edith Beaulieu, Nicolas Lecrenier, Bruce L Innis, Peter Palese, Corey P Mallett, Florian Krammer

**Affiliations:** 1 Department of Microbiology, Icahn School of Medicine at Mount Sinai, New York, NY, USA; 2 Faculty of Life Sciences, University of Vienna, Vienna, Austria; 3 Institute of Molecular Virology, Center of Molecular Biology of Inflammation, University of Münster, Münster, Germany; 4 Graduate School of Biological Sciences, Icahn School of Medicine at Mount Sinai, New York, NY, USA; 5 GSK Vaccines, Laval, QC, Canada; 6 GSK Vaccines, Wavre, Belgium; 7 GSK Vaccines, King of Prussia, PA, USA; 8 Department of Medicine, Icahn School of Medicine at Mount Sinai, New York, NY, USA

## Abstract

Seasonal influenza virus vaccines are generally effective at preventing disease, but need to be well matched to circulating virus strains for maximum benefit. Influenza viruses constantly undergo antigenic changes because of their high mutation rate in the immunodominant haemagglutinin (HA) head domain, which necessitates annual re-formulation and re-vaccination for continuing protection. In case of pandemic influenza virus outbreaks, new vaccines need to be produced and quickly distributed. Novel influenza virus vaccines that redirect the immune response towards more conserved epitopes located in the HA stalk domain may remove the need for annual vaccine re-formulation and could also protect against emergent pandemic strains to which the human population is immunologically naive. One approach to create such universal influenza virus vaccines is the use of constructs expressing chimeric HAs. By sequential immunization with vaccine strains expressing the same conserved HA stalk domain and exotic HA heads to which the host is naive, antibodies against the stalk can be boosted to high titres. Here we tested a monovalent chimeric HA-based prototype universal influenza virus split virion vaccine candidate with and without AS03 adjuvant in primed mice. We found that the chimeric HA-based vaccination regimen induced higher stalk antibody titres than the seasonal vaccine. The stalk antibody responses were long lasting, cross-reactive to distantly related HAs and provided protection *in vivo* in a serum transfer challenge model. The results of this study are promising and support further development of a universal influenza vaccine candidate built on the chimeric HA technology platform.

## Introduction

**Figure 1:**
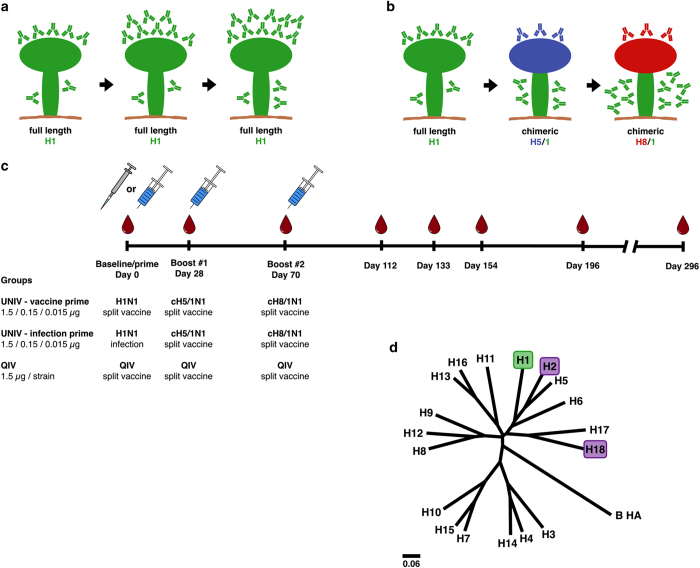
Chimeric haemagglutinin-based universal influenza virus vaccine concept, experimental design and phylogenetic distances of antigens. (**a**) Humans are repeatedly exposed to circulating H1N1 influenza viruses by infection and vaccination. This repeated exposure mainly induces antibodies against the membrane-distal, immunodominant HA head domain. (**b**) By combining exotic HA head domains (pictured in blue and red) with the conserved H1 stalk in the vaccine constructs, the immune response can be (re)directed towards conserved, immuno-subdominant epitopes in the HA stalk. (**c**) The mice were primed with either an H1N1pdm09 virus vaccination or a sublethal H1N1 experimental infection on day 0. This prime was followed by sequential cH5/1N1 split virus vaccination on day 28 and cH8/1N1 split virus vaccination on day 70. The split vaccines were administered in 10-fold dilutions (1.5 to 0.015 μg HA), either with or without AS03 adjuvant. In addition, groups of mice were vaccinated with 1.5 μg HA/strain of either unadjuvanted or AS03 adjuvanted QIV on days 0, 28 and day 70. Mice vaccinated with PBS on days 0, 28 and 70, as well as mice that received a sublethal H1N1 experimental infection prime only were included as controls. The animals were followed for 296 days and bled on days 0, 28, 70, 112, 133, 154, 196 and 296 to assess antibody titres. Ten mice per group were euthanized on day 196 to test their antibodies in a serum transfer challenge experiment. (**d**) The mice were immunized with a vaccine containing the HA stalk domain of H1 (highlighted in green). To test the cross-reactive potential of these antibodies, mouse serum samples were also tested by ELISA against a closely related H2 protein as well as a distantly related H18 protein (both highlighted in purple).

Current seasonal influenza virus vaccines show high vaccine efficacy when they are well matched with circulating virus strains.^1^ However, influenza viruses constantly change their surface glycoproteins that are the targets of most immune responses, which allows them to escape pre-existing immunity, a process called antigenic drift.^2^ Therefore, head-based seasonal influenza virus vaccines have to be re-formulated and re-administered on an annual basis.^3^ In addition, novel viruses can appear at irregular intervals and cause influenza virus pandemics that can claim millions of lives.^4^ Unfortunately, current seasonal influenza virus vaccines are unlikely to protect against future pandemic viruses. Protection from influenza viruses is usually correlated with antibodies that bind to the membrane distal head domain of the haemagglutinin molecule (HA) and inhibit the haemagglutination function (haemagglutination inhibition activity), thereby blocking the virus from attaching to host cell receptors.^5^ However, the head domain has a high plasticity and it is the main site of antigenic drift.^6,7^ The stalk domain is, in contrast to the head domain, relatively conserved but immuno-subdominant (possibly related to lower accessibility of this domain). Several strategies have been developed, which induce broad protection against influenza viruses, overcoming the limitations of currently licensed seasonal vaccines.^8–18^ One of these approaches aims at re-directing the immune response away from the immuno-dominant head domain of the viral HA and towards the more conserved and immuno-subdominant stalk domain using chimeric HAs (cHAs).^8,19,20^ The cHAs are combinations of 'exotic' head domains, mostly from avian influenza virus subtypes to which humans are naive, paired with a conserved stalk domain (e.g., from H1 or H3 HAs).^21,22^ Sequential immunization with cHAs that have different head domains but the same stalk domain can break the immuno-dominance of the head and re-direct the immune response towards the conserved stalk domain (Figures 1a and b). This principle has been demonstrated with experimental vaccines based on recombinant proteins or viral vectors in mice and ferrets.^8,19,20^ Here we assessed whether the same principle holds true using split-vaccine cHAs produced in a pilot process for commercial vaccine production in combination with AS03 adjuvant, a component of a licensed H5N1 pandemic influenza virus vaccine. This work is a necessary precursor to evaluation of the same vaccine strategy in human subjects.

## Results

### Vaccination with cHA-based split vaccines induces stalk-reactive antibodies after inactivated H1N1 vaccination prime

To test the ability of cHA-based vaccines to induce stalk-reactive antibodies, mice were primed with a monovalent pandemic H1N1 vaccine (H1N1pdm09), boosted on day 28 with a cH5/1N1 vaccine and then again on day 70 with a cH8/1N1 vaccine. The prime was given to induce a low level of HA stalk-reactive antibodies that could then be boosted with the chimeric vaccines. The vaccines were given at three different doses (0.015 μg HA, UNIV cHA 0.015; 0.15 μg HA, UNIV cHA 0.15; and 1.5 μg HA, UNIV cHA 1.5) with or without AS03 adjuvant. Control groups included mice that received adjuvanted or non-adjuvanted QIV (at 1.5 μg HA/strain) or phosphate-buffered saline (PBS). The mice were bled on the days of vaccination (0, 28, 70) as well as on day 112, 133, 154, 195 and 296 (Figure 1c) and the serum was analysed for reactivity to the HA stalk (using a cH6/1 HA as antigen). In addition, we assessed reactivity to the heterosubtypic group 1 HAs H2 and H18.

**Figure 2:**
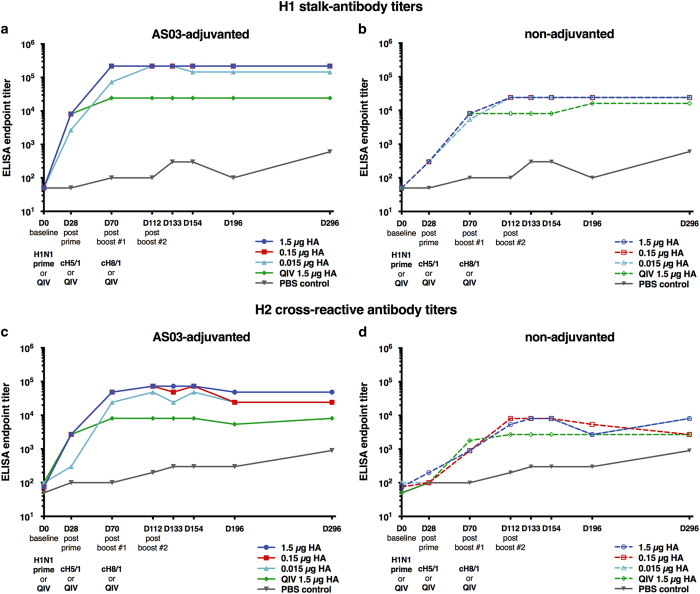

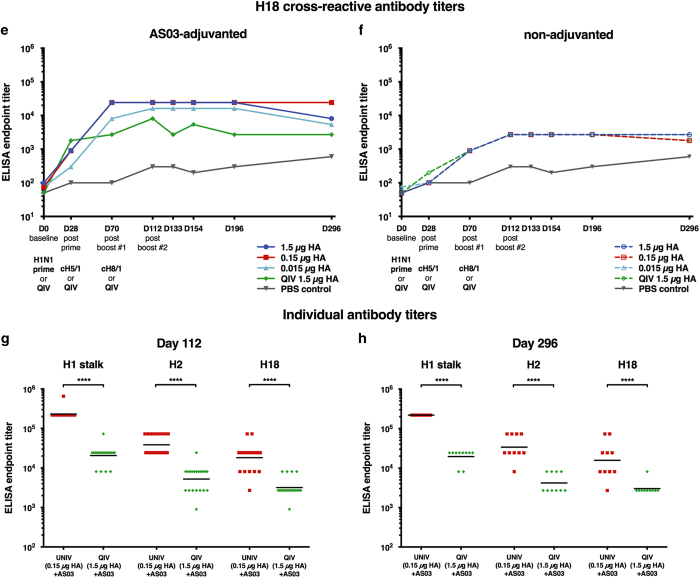
ELISA antibody titres after priming by H1N1pdm09 vaccine. Antibody titres in pooled sera over time are shown against the H1 stalk (**a** and **b**), H2 (**c** and **d**) and H18 (**e** and **f**). Antibody titres of H1N1pdm09 vaccine-primed mice that received adjuvanted UNIV cHA vaccine are consistently higher than titres of mice in the adjuvanted QIV group. AS03 boosted antibody titres against all tested antigens. PBS-vaccinated mice are shown as negative control. (**g**) To assess statistical significance of the differences between UNIV cHA and QIV vaccinated groups, individual sera of the adjuvanted intermediate dose (0.15 μg HA) UNIV cHA group were compared with sera of the adjuvanted QIV (1.5 μg HA/strain) group. On day 112, the titres against the H1 stalk, H2 and H18 proteins were significantly higher in the UNIV cHA group (****P<0.0001) than in the QIV group. (**h**) On day 296, the titres were still significantly higher in the UNIV cHA group against the H1 stalk, H2 and H18 (****P<0.0001, ***P=0.0004, *P=0.0161, respectively). The asterisks refer to the significant zeros after the decimal point of the *p*-value. ^ns^ *P*>0.05; *P≤0.05; **P≤0.01; ***P≤0.001; ****P≤0.0001.

Anti-H1 stalk titres in the adjuvanted groups rose to 1:8,100 on D28 after adjuvanted H1N1 vaccine priming except for the UNIV cHA 0.015 group that only reached a titre of 1:2,700 (Figure 2a). As expected, the AS03 adjuvanted QIV group also attained a titre of 1:8,100 as the vaccine also has a H1N1pdm09 component. Titres of two UNIV cHA groups (1.5 and 0.15 μg) increased to 1:218,700 by day 70 after adjuvanted cH5/1N1 vaccination on day 28 (Figure 2a) and remained at that level for the next 226 days, unchanged by the administration of adjuvanted cH8/1N1 vaccine. In contrast, anti-H1 stalk titres in the low-dose UNIV group (0.015 μg) increased only to 1:72,900 by day 70 after adjuvanted cH5/1N1 vaccine and then to 1:218,700 by day 112 after adjuvanted cH8/1N1 vaccine administered on D70; titres fell 2-fold by day 154 and persisted at that level until day 296 (Figure 2a). The adjuvanted QIV group’s titre increased slightly by D70 to 1:24,300 after receiving a second dose of adjuvanted QIV on D28 and remained at this level (9-fold lower than the peak titres/plateaus of the UNIV groups) for the duration of the experiment despite administration of a third adjuvanted QIV dose.

Next, we tested whether the UNIV candidate induced antibodies against the heterosubtypic H2 and H18 HAs. These two HAs are both group 1 HAs but their phylogenetic distance to H1 is different—with H2 being close to H1 and H18 being distantly related (Figure 1d).^23^ Importantly, H18 lacks a functional sialic acid binding site, which distinguishes it from regular HAs.^24^ Among all groups, the adjuvanted H1N1 vaccine priming dose was less effective in eliciting an anti-H2 response and even less effective in eliciting an anti-H18 response. Accordingly, anti-H2 titres were slightly lower than the anti-H1 stalk titres at subsequent time points. The UNIV 1.5, UNIV 0.15 and UNIV 0.015 groups reached peak titres of 1:72,900, 1:72,900 and 1:42,089 that decreased on day 196 (1.7-fold, 3-fold and 1.7-fold, respectively) and were then maintained at high levels until day 296 (Figure 2c). The peak anti-H2 titre of the UNIV 1.5 group was 3-fold lower than the anti-H1 stalk titre. Vaccination with three doses of adjuvanted QIV at 1.5 μg HA/strain induced even lower anti-H2 titres (1:8,100). The UNIV candidate also elicited anti-H18 reactivity, although it was reduced relative to anti-H2 with titres of 1:24,300, 1:24,300 and 1:14,030 (for the 1.5, 0.15 and 0.015 HA doses, respectively; Figure 2e). Titres in the UNIV 1.5 group were maintained until the termination of the experiment, titres in the two lower doses were slightly falling off after day 196 (Figure 2e). Peak anti-H18 titres in the UNIV 1.5 group were 9-fold lower than the peak anti-H1 stalk titres. Again, vaccination with three doses of adjuvanted QIV (1.5 μg HA/strain) induced a lower anti-H18 titre of 1:8,100.

Finally, to ascertain that the difference between the groups was not driven by a small number of highly responding animals, reactivity of individual mice from the adjuvanted QIV 1.5 μg HA/strain group and the adjuvanted UNIV cHA 0.15 μg group at 196 and 296 days post vaccination was measured. At both the time points, we found very uniform responses (Figures 2g and h). Individual titres were similar to the titres measured in the pools. At both the time points, reactivity in the UNIV cHA 0.15 group was statistically significantly higher than in the adjuvanted QIV 1.5 group for anti-H1 stalk, anti-H2 and anti-H18 antibody titres (Figures 2g and h).

In addition to the AS03-adjuvanted UNIV candidates and QIV control, we also tested non-adjuvanted split vaccines which are known to be poorly immunogenic in naive mice.^25–27^ Reactivity measured in mice that received non-adjuvanted vaccine showed a similar pattern to the reactivity measured in the corresponding adjuvanted groups, but in general, responses were approximately 9-fold lower (Figures 2b, d and f).

### cHA vaccines boost infection-induced pre-existing stalk-reactive antibodies

**Figure 3:**
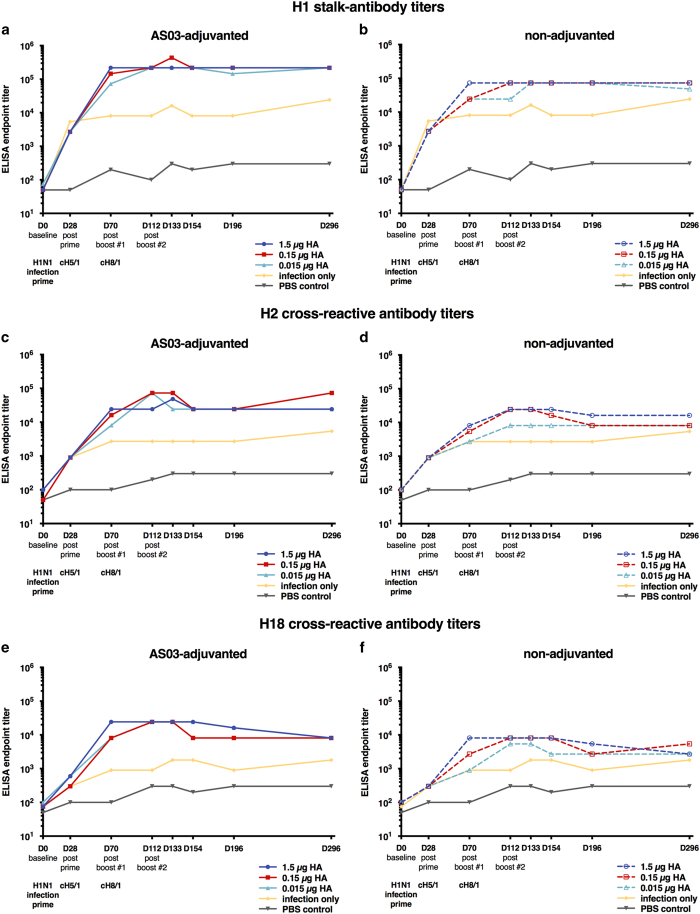
ELISA antibody titres after priming by H1N1pdm09 infection. Antibody titres over time are shown against the H1 stalk (**a** and **b**), H2 (**c** and **d**) and H18 (**e** and **f**) after mice were primed via sublethal H1N1pdm09 infection. The mice that received an infection prime only, as well as PBS-vaccinated mice were added as controls. Antibody titres after unadjuvanted vaccination were generally lower—but higher than in the vaccine prime shown in Figure 2—and a more pronounced dose response effect was observed.

The majority of the global population is first exposed to influenza virus antigens by natural infection. It is known that natural infection with influenza viruses induces stalk-reactive antibodies.^28–30^ Here we wanted to investigate how well cHA-based split vaccines would boost stalk-reactive antibodies in animals primed by experimental influenza virus infection, which is a model for natural infection in humans. The mice were primed by infection with H1N1pdm09 virus at a sublethal dose and then sequentially vaccinated with the UNIV candidates (cH5/1N1 and cH8/1N1 at different doses). An infection-only group and a PBS group were the controls. Infection with H1N1pdm09 induced an anti-H1 stalk titre of 1:8,100 and the titre remained at this level with minor fluctuation until the end of the experiment at day 296 (Figure 3a). Vaccination with all three doses of UNIV boosted anti-H1 stalk titres to a peak/plateau of 1:218,700, a titre similar to the titre observed in adjuvanted H1N1 vaccination-primed mice (Figure 3a and Figure 2a). Again, the second UNIV cHA vaccination on day 70 appeared to have minimal effect (Figure 3a). We also assessed reactivity to H2 and H18 in these groups. Experimental infection with H1N1pdm09 virus induced low (1:2,700) anti-H2 titres that could be boosted by vaccination with cHAs (Figure 3c). All the three doses had a similar effect on reactivity with peak anti-H2 titres of 1:72,900 on day 112 (1:24,000 for UNIV cHA 1.5; Figure 3c). However, in this case, a boost caused by the second cHA vaccination was observed. An H1N1pdm09 infection primed low-level anti H18 titres (1:900; Figure 3e). Vaccination with UNIV cHA 1.5, 0.15 and 0.015 boosted anti-H18 titres to a peak of 1:24,300 on day 112. Although the second cHA vaccination with UNIV cHA 1.5 on day 70 appeared to have no effect, anti-H18 titres increased after the second vaccination with UNIV cHA 0.15 and 0.015.

The experiment was also performed with non-adjuvanted vaccines. As described above, the peak/plateau titres were lower when non-adjuvanted vaccines were used and a more apparent dose response was observed for H2 and H18 cross-reactive titres (Figure 3b, d and f). However, the difference in peak titres between adjuvanted and non-adjuvanted UNIV cHA groups was smaller (approximately 3-fold) when mice were primed by experimental H1N1pdm09 infection compared with mice primed with H1N1pdm09 vaccine (approximately 9-fold difference, as described above).

### cHA vaccines boost anti-neuraminidase antibodies

**Figure 4:**
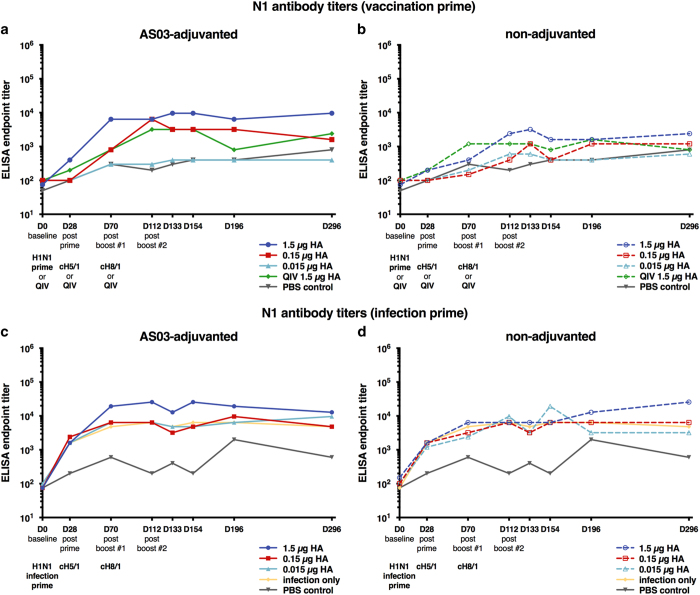
ELISA antibody titres against H1N1pdm09 neuraminidase. Antibody titres over time are shown against the H1N1 neuraminidase after mice were primed with either H1N1pdm09 vaccine (**a** and **b**) or sublethal H1N1pdm09 infection (**c** and **d**). Antibodies against the neuraminidase were generally low and a clear dose response effect was observed for the UNIV cHA groups. Only the highest dose UNIV cHA vaccine induced consistently high NA antibody titres.

Induction of anti-NA antibodies is usually not assessed after vaccination with seasonal influenza virus vaccines and no correlation between NA-specific antibody titres and protection has been formally established yet. However, there are many reports that indicate that antibodies to the NA can contribute substantially to protection.^17,31–37^ To characterize the anti-NA response to the UNIV candidate, we performed quantitative enzyme-linked immunosorbent assays (ELISAs) using recombinant tetrameric N1 (H1N1pdm09) as the antigen. Titres in the adjuvanted UNIV cHA 1.5 group increased after the H1N1pdm09 prime and after the cH8/1 boost and reached a plateau at 1:8,100 that was maintained until the end of the experiment (Figure 4a). The UNIV cHA 0.15 and 0.015 candidates were less immunogenic, with UNIV cHA 0.15 peaking at 1:6,400 at day 112 and slowly declining over time and UNIV cHA 0.015 showing no reactivity above that of the PBS control demonstrating a dose response for the N1 antigen. Adjuvanted QIV administered thrice was similarly immunogenic to UNIV 0.15, whereas the UNIV 1.5 regimen was approximately 3-fold more immunogenic than the adjuvanted QIV (also 1.5 μg of purified HA; Figure 4a). Similar results were obtained with non-adjuvanted vaccines albeit at approximately 3-fold lower peak titres (Figure 4b). Interestingly, priming by experimental infection induced moderate anti-NA titres, which could be boosted 16-fold to 1:25,600 by the highest adjuvanted UNIV (1.5 μg) regimen (Figure 4c). All other vaccines failed to boost anti-NA titres above the level of the experimental infection prime (Figures 4c and d). We also tested reactivity to an N3 NA antigen. Titres were low and no statistical difference between the different groups was observed (data not shown).

### Transfer of sera containing anti-H1 stalk antibodies reduces virus replication in recipient mice

**Figure 5:**
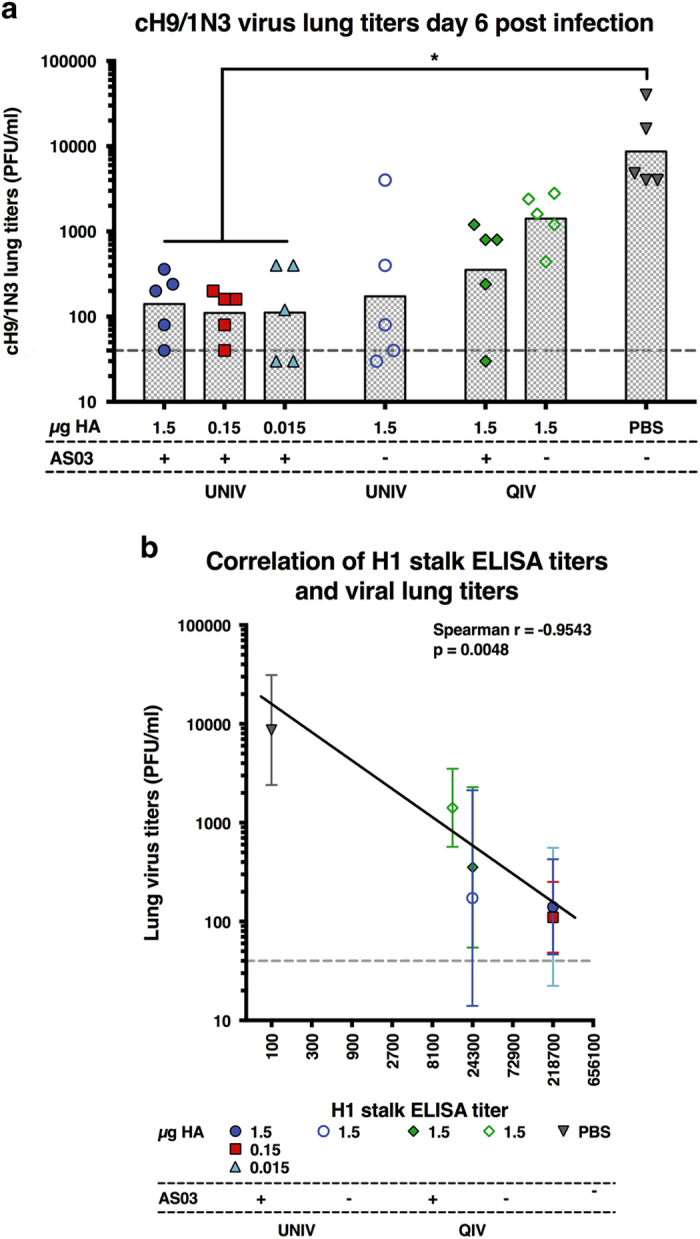
Viral lung titres after serum transfer and cH9/1N3 virus challenge. To assess *in vivo* protective activity of anti-stalk antibodies, sera harvested from 10 mice on day 196 were transferred into naive mice, which were then challenged with the cH9/1N3 virus. (**a**) Viral lung titres were significantly lower only in mice that received serum from the AS03-adjuvanted UNIV cHA groups as compared with titres in mice that received serum from the PBS control group (UNIV cHA 1.5 μg HA *P*=0.0416, UNIV cHA 0.15 μg HA *P*=0.0149, UNIV cHA 0.015 μg HA *P*=0.0176). Transfer of serum from animals vaccinated with QIV with and without AS03 adjuvant, as well as UNIV cHA 1.5 μg HA without adjuvant resulted in moderate reduction in viral titres. (**b**) ELISA antibody titres against the H1 stalk are negatively correlated with viral lung titres (Spearman *r*=−0.9543, *P*=0.0048). This further supports the hypothesis that the protection from viral infection in the experiment was conferred by stalk antibodies. The asterisks refer to the significant zeros after the decimal point of the p-value. Symbol meaning ^ns^ *P*>0.05; *P≤0.05; **P≤0.01; ***P≤0.001; ****P≤0.0001.

Next, we assessed whether the anti-stalk antibodies induced by sequential vaccination with cHAs have biological activity *in vivo* in a serum transfer/challenge approach.^38^ Ten mice from the three AS03-adjuvanted UNIV cHA groups, the non-adjuvanted UNIV cHA 1.5 group, the adjuvanted and the non-adjuvanted QIV groups, and the PBS control group were terminally bled on day 196. Sera were pooled within groups and then transferred into naive mice. Two hours post transfer, these mice were challenged with a cH9/1N3 virus that expresses an H9 head domain in combination with an H1 stalk domain and a N3 neuraminidase.^28,39,40^ As sera from sequentially vaccinated mice will only react to the H1 stalk domain but not to the H9 head^39^ and the N3 NA, this virus allows the assessment of protection conferred by anti-stalk antibodies only.^39^ On day 6 post challenge, the mice were killed, lungs were harvested and viral titres were measured in a plaque assay. The mice that were administered serum from the PBS control group had lung virus titres in the range of 10^4^ plaque-forming unit (PFU)/ml (geometric mean titre of 8,676 PFU/ml, Figure 5a). The mice administered serum from recipients of non-adjuvanted QIV and adjuvanted QIV showed reduced geometric mean virus lung titres of 1,415 PFU/ml and 354 PFU/ml, respectively. The animals that were administered serum from recipients of non-adjuvanted cHA vaccine (UNIV cHA 1.5) showed even lower geometric lung titres of 173 PFU/ml but the individual titres were relatively non-homogeneous. Geometric mean titres for all animals that were administered serum from recipients of adjuvanted UNIV cHA groups were low and homogeneous (141, 110, 111 PFU/ml). Importantly, the reduction of viral lung titres compared with the PBS control group was only significant in the AS03-adjuvanted UNIV cHA groups. Furthermore, the serum anti-stalk titre measured by ELISA correlated inversely with lung virus titres (Spearman *r*=−0.9543, *P*=0.0048, Figure 5b).

## Discussion

Chimeric HA-based universal influenza vaccines aim at inducing high levels of broadly protective antibodies by re-directing the immune response to the conserved stalk domain and by breaking the immunodominance of the head domain.^8,41^ Although the overall concept has been proven in animal models using experimental vaccine platforms like DNA vaccination and virus-vectored vaccines,^19,20,41–44^ we wanted to explore whether split virus vaccines produced using an industrial pilot scale production process would support this approach as well. This question is important to investigate as a positive result would support evaluation of the cHA vaccines in human subjects. Here we demonstrate that cHA-based split vaccines induce stalk-reactive antibodies in primed mice. As hypothesized, sequential vaccination with cHA induced a higher IgG antibody titre than vaccination with three doses of QIV. The adjuvant had an important role in inducing high anti-stalk IgG antibody titres and the titres achieved with non-adjuvanted split vaccine were significantly lower.^45^ However, it is important to mention that non-adjuvanted split vaccines have previously been found to be poorly immunogenic in murine and ferret models compared with whole virus, most likely due to the absence of significant amounts of pathogen-associated molecular patterns.^25–27,46^ Importantly, although non-adjuvanted cHA-based vaccines did not induce very high titres in vaccine-primed mice, they efficiently boosted stalk IgG antibodies in animals primed by experimental infection.

Furthermore, we noticed that anti-stalk antibody levels reached a titre of 1:218,700 and it could not be boosted any higher. Mice typically have relatively low total IgG serum concentrations of 1–2 mg/ml (as compared with humans who have 13 mg/ml on average).^47^ Under the assumption that all present IgG was stalk-reactive (which is not the case, only a proportion of the serum IgG will react with the stalk domain), a measured titre of 1:218,700 would translate into a minimum binding concentration of 4.6–9.1 ng/ml for stalk-reactive antibodies. It has been shown that human stalk-reactive antibodies have similar minimum binding concentrations, which means that the observed plateau might be a consequence of the lower IgG serum concentrations in the mouse model.^48^ In fact, serum from mice that received the lowest tested cHA dose (0.015 μg HA) did not reach this plateau after priming and one dose of cHA vaccine; however, the second cHA vaccine dose did boost antibody titres.

Anti-stalk antibodies have been shown to cross-react and cross-neutralize different HA subtypes, mostly within the same HA group.^47,49–51^ However, it is important to keep in mind that not all induced stalk-reactive antibodies will cross-react and that some antibodies will have more narrow reactivity patterns than others. In our study, reactivity was highest towards H1, followed by H2 (approximately 3-fold lower) and H18 (approximately 9-fold lower). Interestingly this also reflects the phylogenetic distance between these three HAs (Figure 1d). This lower reactivity to HAs that are more distantly related to the HA stalk used for vaccination is expected and has been observed previously but the level of antibody reached might still afford full protection against heterosubtypic challenge.^9,10^ However, cross-reactivity between group A1 and group A2 HA stalk domains is low, which makes it necessary to also develop a group A2 universal vaccine component^8–10,47^ (in addition to a conserved B virus constituent) as part of a multivalent universal influenza virus vaccine.

In addition to anti-stalk antibodies, the cHA-based vaccine also induced robust anti-NA titres when given with the AS03 adjuvant at the highest dose. Interestingly, the adjuvanted high-dose cHA vaccine was also the only treatment in our study that boosted titres of anti-NA antibodies induced by experimental infection. This is important as the majority of the global population is first exposed to influenza virus antigens via natural infection. However, this is only relevant for viruses expressing N1 and N2 NAs as no pre-existing immunity induced by natural infection exists in the population against N3–N9 NAs.

Finally, our results demonstrate that anti-stalk titres can be maintained at high levels over a long period of time, at least in the mouse model. This is highly important since a universal influenza vaccine that induces only short-term immunity would be of limited use. The data reported here support the further development of cHA-based broadly protective influenza virus constructs and pave the way for future clinical evaluation of these vaccine candidates.

## Materials and Methods

### Vaccines, viruses and adjuvants

The investigational monovalent cH5/1N1 and cH8/1N1 egg-derived, inactivated, split virion vaccines were produced from cH5/1_Cal09_N1_Cal09_ and cH8/1_Cal09_N1_Cal09_ reverse genetics viruses as described previously.^22,52^ The H5 head domain of cH5/1_Cal09_N1_Cal09_ virus was derived from the HA of A/Vietnam/1203/04 (H5N1), the head domain of the cH8/1_Cal09_N1_Cal09_ virus was derived from the HA of A/mallard/Sweden/24/02 (H8N4), the stalk domain and NA of both viruses was derived from A/California/04/09 (H1N1) and the internal genes were derived from the high-yielding vaccine donor strain A/Puerto Rico/8/34 (H1N1). The cH5/1 and cH8/1 antigens were influenza-split virions produced by GSK (Sainte-Foy, QC, Canada). The HA content of these vaccines was determined by densitometry of Coomassie blue-stained SDS-PAGE gels. The monovalent H1N1 egg-derived, inactivated, split virion vaccine was produced from A/California/7/2009 (H1N1) NYMC X-179A reassortant virus generated using classical reassortant methodology by GSK. The HA content of this vaccine was determined by single radial immuno-diffusion assay using reagents from Center for Biologics Evaluation and Research (Food and Drug Administration, MD, USA) or National Institute for Biological Standards and Control (Hertfordshire, UK). The seasonal quadrivalent inactivated, split virion vaccine was the 2011 Northern Hemisphere vaccine that was produced from A/California/7/2009 (H1N1) NYMC X-179A and A/Victoria/210/2009 (H3N2) NYMC X-187 reassortant viruses generated using classical reassortant methodology as well as B/Brisbane/60/2008 (B/Victoria lineage) and B/Florida/4/2006 (B/Yamagata lineage) wild-type viruses. The HA content of the seasonal quadrivalent vaccine was determined by single radial immuno-diffusion assay using reagents from Center for Biologics Evaluation and Research or National Institute for Biological Standards and Control. All vaccines used in this study were manufactured in embryonated eggs by GSK Vaccines. The A/California/7/2009 (H1N1) wild-type virus (kindly supplied by Xiyan Xu, Influenza Division, Centers for Disease Control and Prevention, Atlanta, GA, USA) was propagated on Madin-Darby Canine Kidney cells before priming the mice. AS03 was manufactured by GSK Vaccines.^53,54^ AS03_B_ is defined as an Adjuvant System containing α-tocopherol and squalene in an oil-in-water emulsion (5.93 mg tocopherol/dose). The AS03 used for the studies described herein is equivalent to 1/10th of the adult human dose of AS03_B_, and hereafter is referred to as AS03. The split virion vaccines were ad-mixed with the AS03 by gentle inversion preceding each immunization.

### Immunogenicity study design and treatment schedule

All mouse procedures were approved in advance by the Institutional Animal Care Committee at the Institut Armand Frappier (Laval, QC, Canada) according to the guidelines of the Canadian Council on Animal Care. Six- to eight-week-old female BALB/c mice (Charles River, Saint-Constant, QC, Canada) were randomly assigned to a treatment group (*n*=20 mice per group). The mice were first primed intranasally with live A/California/7/2009 (H1N1) wild-type virus (10^6^ tissue culture infectious dose 50) or intramuscularly with a 100-fold dose range of the H1N1pdm09 vaccine (1.5 to 0.015 μg HA) ±AS03 on study day 0. The virus and vaccine primed mice were then serially immunized intramuscularly with a 100-fold dose range of the monovalent detergent-split cH5/1 and cH8/1 universal influenza vaccines (UNIV, 1.5 to 0.015 μg HA) ±AS03 on study days 28 and 70. Additional mice (not primed with H1N1pdm09 virus or split vaccine previously) were serially immunized intramuscularly with the seasonal quadrivalent vaccine (QIV, 1.5 μg HA/strain) ±AS03 on study days 0, 28 and 70. The mice vaccinated with PBS on days 0, 28 and 70 were included as negative controls for both the infection and intramuscular prime experiments. Furthermore, an infection prime-only control group was included as well. The mice were bled on study days 0, 28, 70, 112, 133, 154, 196 and 296 as shown in Figure 1c, and the serum IgG antibody titres to cH6/1 HA (group 1 stalk) as well as H2 and H18 full-length HA were determined by ELISA.

### Serum transfer study design

All the mouse procedures were approved in advance by the Institutional Animal Care and Use Committee at the Icahn School of Medicine at Mount in accordance with the Animal Act PL99–158 (as amended) and guidelines stated in the ‘Guide for the Care and Use of Laboratory Animals’. Six-to-eight-week-old female BALB/c mice (The Jackson Laboratory, Bar Harbor, ME, USA) were randomly assigned to a treatment group. Ten mice from each group in the immunization study were terminally bled on day 196 post prime vaccination. Serum pools were created for all groups that received the inactivated virus prime, except for the groups that received the lower doses (0.15 μg HA and 0.015 μg HA) of non-adjuvanted chimeric HA. As a negative control, a pool of serum from PBS-vaccinated mice was included as well. Two hundred and fifty microlitres of pooled serum were transferred into five mice per group. Two hours after the serum transfer, the mice were challenged with 10^5^ PFUs of chimeric H9/1N3 virus. This virus has the exotic head of an HA and neuraminidase to which the vaccinated mice are immunologically naive, but their serum will recognize the conserved H1 stalk domain of the virus. Therefore, only H1 stalk-reactive antibodies can contribute to protection in this experiment. On day 6 post challenge, the mice were killed and their lungs extracted. The lungs were homogenized, cell debris removed by centrifugation and supernatants frozen at −80 °C until further use. Virus titres in the lung supernatants were measured by plaque assay as previously described.^28,39^

### ELISA methodology

The recombinant cH6/1 (head domain from HA of H6N1 virus A/mallard/Sweden/81/02, stalk domain from HA of H1N1 virus A/Puerto Rico/8/34), H2 (A/mallard/Netherlands/5/99), H18 (A/flat-faced bat/Peru/033/10), N1 (A/California/04/09) and N3 (A/swine/Missouri/4296424/06) proteins that were used as ELISA substrates were produced using the baculovirus expression system in insect cells as described previously.^55,56^ The serum samples were pooled for ELISA analysis and tested in technical duplicates. In addition, to test statistical significance of the differences in H1 stalk antibody titres, individual serum samples were tested for reactivity on day 112 and day 296 post prime for the AS03-adjuvanted QIV group and the AS03-adjuvanted, second highest dose (0.15 μg HA) chimeric HA group. High-binding 96-well ELISA plates (Thermo Scientific, Waltham, MA, USA) were coated with 50 μl of antigen diluted at a concentration of 2 μg/ml in coating buffer (50 mM sodium carbonate, 50 mM sodium hydrogen carbonate, pH 9.4). The plates were incubated for 12–18 h at 4 °C and the coating solution was removed with three washes of PBS-T (PBS, 0.1% Tween-20, pH 7.4). The plates were blocked with 220 μl of blocking solution (PBS-T with 3% milk powder) per well for 1 h at room temperature. The blocking solution was replaced with 100 μl of fresh blocking solution per well. For 3-fold dilutions, additional 40 μl of blocking solution was added to the first well of each dilution series (90 μl for 2-fold dilutions). Ten microlitres of pre-diluted serum was added to the first well of each dilution series. Fifty microlitres were transferred for 3-fold serial dilutions (100 μl for 2-fold dilutions). Two columns per plate did not contain any serum and were used as blanks. The plates were incubated for 2 h at room temperature and then washed three times with PBS-T. Horse radish peroxidase-labelled anti-mouse IgG (whole molecule, Sigma, St. Louis, MO, USA) was used as secondary antibody. Fifty microlitres of secondary antibody diluted at a concentration of 1:3,000 in blocking solution was added to each well and the plates were incubated for 1 h at room temperature. The plates were washed four times and developed with 100 μl of SigmaFast OPD (Sigma) substrate per well for 10 min. Enzymatic colour development was stopped with 50 μl of 3 M hydrochloric acid per well and the plates were read at an absorbance of 490 nm. The results are reported as end-point titres. The end-point titre was defined as the last dilution in which the reactivity of a serum sample was still above the cut-off of the average of the blanks plus three standard deviations.

### Data analysis

Data were analysed using Microsoft Excel and Graphpad Prism software. Statistical differences for ELISA titres were calculated with unpaired *t*-tests. Day 6 virus lung titres were compared with a Kruskal–Wallis test and a Dunn’s multiple comparisons test. Correlation of day 6 virus lung titres and H1 stalk antibody titres was calculated by nonparametric Spearman correlation, and a nonlinear log–log line fit was plotted.
